# Analytical study of *RUNX1-RUNXT1*, *PML-RARA*, *CBFB-MYH11*, *BCR-ABL1^p210^*, and *KMT2-MLLT3* in Mexican children with acute myeloid leukemia: A multicenter study of the Mexican interinstitutional group for the identification of the causes of childhood leukemia (MIGICCL)

**DOI:** 10.3389/fped.2022.946690

**Published:** 2022-11-14

**Authors:** Omar Sepúlveda-Robles, Elva Jiménez-Hernández, Victoria Domínguez-Catzín, Eber Gómez-Flores, Jorge Alfonso Martín-Trejo, Janet Flores-Lujano, José Refugio Torres-Nava, Juan Carlos Núñez-Enríquez, Marlon De Ita, Aurora Medina-Sanson, Minerva Mata-Rocha, Blanca Angelica Morales-Castillo, Juan Carlos Bravata-Alcántara, Alan Steve Nájera-Cortés, Norberto Sánchez-Escobar, José Gabriel Peñaloza-Gonzalez, Rosa Martha Espinosa-Elizondo, Luz Victoria Flores-Villegas, Raquel Amador-Sanchez, Darío Orozco-Ruiz, Maria Luisa Pérez-Saldívar, Martha Margarita Velázquez-Aviña, Laura Elizabeth Merino-Pasaye, Karina Anastacia Solís-Labastida, Ana Itamar González-Ávila, Jessica Denisse Santillán-Juárez, Vilma Carolina Bekker-Méndez, Silvia Jiménez-Morales, Angélica Rangel-López, Haydeé Rosas-Vargas, Juan Manuel Mejía-Aranguré

**Affiliations:** ^1^ Unidad de Investigación Médica en Genética Humana, UMAE Hospital de Pediatría, Centro Médico Nacional “Siglo XXI”, Instituto Mexicano del Seguro Social (IMSS), Mexico City, Mexico; ^2^ Servicio de Hematología Pediátrica, Hospital General “Gaudencio González Garza”, Centro Médico Nacional “La Raza”, Instituto Mexicano del Seguro Social (IMSS), Mexico City, Mexico; ^3^ Genes & Care, Centro de Diagnóstico Molecular, Mexico City, Mexico; ^4^ Servicio de Hematología Pediátrica, UMAE Hospital de Pediatría, Centro Médico Nacional “Siglo XXI”, Instituto Mexicano del Seguro Social (IMSS), Mexico City, Mexico; ^5^ Unidad de Investigación Médica en Epidemiología Clínica, UMAE Hospital de Pediatría, Centro Médico Nacional “Siglo XXI”, Instituto Mexicano del Seguro Social (IMSS), Mexico City, Mexico; ^6^ Servicio de Oncología, Hospital Pediátrico de Moctezuma, Secretaría de Salud de la Ciudad de México, Mexico City, Mexico; ^7^ Servicio de Hemato-Oncología, Hospital Infantil de México Federico Gómez, Secretaría de Salud (SSa), Mexico City, Mexico; ^8^ Laboratorio de Genética y Diagnóstico Molecular, Hospital Juárez de México, Secretaría de Salud (SSa), Mexico City, Mexico; ^9^ Facultad de Medicina y Cirugía, Universidad Autónoma “Benito Juárez” de Oaxaca, Oaxaca City, Mexico; ^10^ Servicio de Onco-Pediatría, Hospital Juárez de México, Secretaría de Salud (SSa), Mexico City, Mexico; ^11^ Servicio de Hematología Pediátrica, Hospital General de México, Secretaría de Salud (SSa), Mexico City, Mexico; ^12^ Servicio de Hematología Pediátrica, Centro Médico Nacional “20 de Noviembre”, Instituto de Seguridad Social al Servicio de los Trabajadores del Estado (ISSSTE), Mexico City, Mexico; ^13^ Hospital General Regional No. 1 “Carlos McGregor Sánchez Navarro”, Instituto Mexicano del Seguro Social (IMSS), Mexico City, Mexico; ^14^ Servicio de Hemato-Oncología Pediatrica, Hospital Regional 1° de Octubre, Instituto de Seguridad Social al Servicio de los Trabajadores del Estado (ISSSTE), Mexico City, Mexico; ^15^ Unidad de Investigación Médica en Inmunología e Infectología, Hospital de Infectología “Dr. Daniel Méndez Hernández”, Centro Médico Nacional “La Raza”, Instituto Mexicano del Seguro Social (IMSS), Mexico City, Mexico; ^16^ Laboratorio de Genómica del Cáncer, Instituto Nacional de Medicina Genómica, Mexico City, Mexico; ^17^ Coordinación de Investigación en Salud, Instituto Mexicano del Seguro Social (IMSS), Mexico City, Mexico; ^18^ Facultad de Medicina, Universidad Nacional Autónoma de México (UNAM), Mexico City, Mexico

**Keywords:** AML – acute myeloid leukaemia, fusion genes, translocation, pediatric population, Mexican population

## Abstract

**Background:**

The distribution of *RUNX1-RUNXT1*, *PML-RARA*, *CBFB-MYH11*, *BCR-ABL1^p210^*, and *KMT2A-MLLT3* in the pediatric population with acute myeloid leukemia (AML) in many countries of Latin America is largely unknown. Therefore, we aimed to investigate the frequency of these fusion genes in children with *de novo* AML from Mexico City, which has one of the highest incidence rates of acute leukemia in the world. Additionally, we explored their impact in mortality during the first year of treatment.

**Methods:**

We retrospectively analyzed the presence of *RUNX1-RUNXT1*, *PML-RARA*, *CBFB-MYH11*, *BCR-ABL1^p210^*, and *KMT2A-MLLT3* by RT-PCR among 77 patients (<18 years) diagnosed with *de novo* AML between 2019 and 2021 in nine Mexico City hospitals.

**Results:**

The overall frequency of the fusion genes was 50.7%; *RUNX1-RUNXT1* (22.1%) and *PML-RARA* (20.8%) were the most prevalent, followed by *CBFB-MYH11* (5.2%) and *BCR-ABL1^p210^* (2.4%). *KMT2A-MLLT3* was not detected. Patients with *PML-RARA* showed the lowest survival with high early mortality events. However, more studies are required to evaluate the impact of analyzed fusion genes on the overall survival of the Mexican child population with AML.

**Conclusion:**

The pediatric population of Mexico City with AML had frequencies of *AML1-ETO*, *PML-RARA*, *CBFB-MYH11*, and *BCR-ABL1^p210^* similar to those of other populations around the world. Patients with *BCR-ABL1^p210^*and *CBFB-MYH11* were few or did not die, while those with *MLL-AF9* was not detected. Although patients with *PML-RARA* had a low survival and a high early mortality rate, further studies are needed to determine the long-term impacts of these fusion genes on this Latino population.

## Introduction

The Acute myeloid leukemia (AML) represents ∼20% of acute leukemia cases in children and 80% in adults (1). Although AML in children and adolescents does not affect a great number of patients, its lethality is the highest amongst those of acute leukemias, with a global 5-year net survival of ∼42.9% and a maximum achievable survival of 73.1% (2). Among Hispanics, the population of Mexico City has one of the highest incidence rates, with an adjusted average annual incidence rate of 8.18 per million for children under the age of 15 years, which has been increasing for the last 10 years (3, 4).

The five most common chromosomal translocations in patients with AML are t(8;21), t(15;17), inv(16), der(11), and t(9;22), which encode the fusion genes *RUNX1-RUNXT1* (*AML1-ETO*), *PML-RARA*, *CBFB-MYH11*, *MLL-*fusions, and *BCR-ABL1*, respectively (5). The identification of which is useful for stratifying patients, revealing prognoses, and making decisions regarding treatment protocols (5). The frequency of this fusion genes in pediatric patients is ∼50%, which is much higher than that in adults (6–9), and their individual frequencies range from 3 to 20% (5). Patients with *AML1-ETO*, *PML-RARA*, and *CBFB-MYH11* tend to have favorable outcomes; therefore, they are not recommended for stem cell transplantation at first complete remission (5). By contrast, patients who harbor *MLL* fusion genes tend to have intermediate to poor prognoses (10). Importantly, the WHO classification categorizes the specific fusion gene *MLL-AF9* as an entity and recommends that the partners in the variant *MLL* fusion genes should be identified; however, *MLL-AF9* is one of the most common fusion gene presented in patients with AML (10, 11). The presence of *BCR-ABL1* in *de novo* AML cases appears to be a rare disease subtype (0.5%–3%) that is apparently related to induction failures and relapses (12).

Despite its manifest clinical relevance, the distribution of these fusion genes in the pediatric population in Mexico with AML is largely unknown. Studies performed in Mexican patients have been conducted with small population sizes or have been limited to specific fusion genes, such as *AML1-ETO* or *PML-RARA* (13–15). Regarding *CBFB-MYH11*, *MLL* fusions, or *BCR-ABL1*, there are no data, or the few studies performed have considered patients from individual hospitals without population-level information (13–16). Given the high incidence of AML in Mexican children, as well as the importance of knowing the epidemiological behavior of *AML1-ETO*, *PML-RARA*, *CBFB-MYH11*, *MLL-AF9*, and *BCR-ABL1^p210^* for diagnosis and best treatment selection, the aims of this multicenter study performed by the Mexican Interinstitutional Group for the Identification of the Causes of Childhood Leukemia (MIGICCL) were to determine the frequencies of these five common fusion genes in children with AML from Mexico City. Additionally, we explored their impact in mortality during the first year of treatment.

## Materials and methods

### Patients, hospitals, and clinical data

A total of 77 children under 18 years old newly diagnosed with AML were recruited between January 2019 and June 2021, from nine hospitals belonged to four Mexican Health Institutions: (1) Secretaría de Salud del Distrito Federal (Hospital Pediátrico de Moctezuma), (2) Instituto de Seguridad Social al Servicio de los Trabajadores del Estado (Hospital Regional No. 1 de Octubre and Centro Médico Nacional 20 de Noviembre), (3) Secretaría de Salud (Hospital General de México Dr. Eduardo Liceaga, Hospital Infantil de México Federico Gómez, and Hospital Juárez de México), and (4) Instituto Mexicano del Seguro Social (Hospital de Pediatría, Centro Médico Nacional Siglo XXI, Centro Médico Nacional La Raza, and Hospital General Regional No. 1 Carlos Mac Gregor Sánchez Navarro). Information regarding sex, age, white blood cell (WBC) count at diagnosis, morphological subtype, and immunophenotype was collected from the patients' clinical records. Patients were treated with protocols PETHEMA-APL05 (*n* = 13), NOPHO-AML93 (*n* = 18), BFM-2001 (*n* = 20), or BFM-1998 (*n* = 26), were categorized into the age groups: infants <2 years; children: 2 to <13 years; and adolescents: 13 to <18 years (17).

### RNA isolation, cDNA synthesis, and fusion gene detection by PCR

Total RNA was isolated from mononuclear cells using Direct-zol RNA MiniPrep kit (Zymo Research), and its quality was confirmed by visualization of intact 28S and 18S bands in agarose gels. Reverse transcription reaction was carried out using 500 ng of total RNA and the iScript Reverse Transcription Supermix (Bio-Rad). All PCR reactions were performed using Multiplex TEMPase 2× Master Mix (Ampliqon), using a start incubation step at 95°C for 15 min, and 35 cycles. Then, reactions for *AML1-ETO*, *PML-RARA^(bcr1, bcr2)^*, and *CBFB-MYH11* were incubated at 94°C/45 s, 63°C/1 min, 72°C/1:30 min, 72°C/5 min; *BCR-ABL1^p210^* was incubated at 96°C/30 s, 60°C/45 s, 72°C/1 min, 72°C/5 min; *MLL-AF9* was incubated at 94°C/1 min, 60°C/1 min, 72°C/1 min, 72°C/5 min. *PML-RARA^(bcr3)^* was detected in a separately reaction with incubation at 94°C/30 s, 65°C/1 min, 72°C/1 min, 72°C/5 min. Oligonucleotide sequences described previously were used for detection of *AML1-ETO* (Fw-ctaccgcagccatgaagaacc, Rv-agaggaaggcccattgctgaa), *CBFB-MYH11* (Fw-gcaggcaaggtatatttgaagg, Rv-tcctcttctcctcattctgctc), *BCR-ABL1^p210^* (Fw-acagaattccgctgaccatcaataag, Fv-tgttgactggcgtgatgtagttgcttgg), *MLL-AF9* (Fw-ctcagccacctactacaggac, Fv-agcgagcaaagatcaaaatc), *PML-RARA^(bcr1, bcr2)^* (Fw-cagtgtacgccttctccatca, Rv-gcttgtagatgcggggtaga) (18–20), and *PML-RARA^(bcr3)^* (Fw-ctgctggaggctgtggac, Rv-gcttgtagatgcggggtaga) (21). The PCR products were analyzed by agarose gel electrophoresis: *AML1-ETO* (395 bp), *PML-RARA^(bcr1, bcr2, bcr3)^* (381, 345 and 376 bp), *CBFB-MYH11* (418 bp), *BCR-ABL1^P210^* (310 bp), and MLL-AF9 (314 bp) (Supplementary Figure S1); and sequenced for validation using a 3500 Genetic Analyzer from Applied Biosystems (Supplementary Figure S2). Cell lines or validated positive patient samples were used as positive controls. A blank control without cDNA was used in each PCR.

### Statistical analysis and ethical statement

The frequencies of the fusion genes were calculated using SPSS IBM V.21. The study was conducted according to the guidelines of the Declaration of Helsinki and approved by the Institutional Research and Ethics Committee of the Centro Médico Nacional Siglo XXI (project number: R-2015-785-121). The patients/participants provided their written informed consent to participate in this study.

## Results

### Demographic and biological characteristics of studied population

We registered 77 patients (<18 years) with confirmed diagnoses of AML in nine public hospitals between January 2019 and June 2021. We found that 64.9% of the patients were male and 35.1% were female, with a median age of 9.4 years (Table 1). The largest age group was children, representing 55.8%, followed by adolescents (35.1%) and infants (9.1%) (Table 1). The mean WBC was 67.96 × 10^9^/L, with a range of 0.76–500 × 10^9^/L. According to the French–American–British (FAB) classification, 29.9% of the patients had the M2 or M4 subtype and 18.2% had M3; together, these three subtypes accounted for 78% of the patients (Table 1). The remaining few patients (*n* = 11, 14.28%) were classified as M0, M1, M5, and M6 (Table 1).

**Table 1:** Characteristics of pediatric patients diagnosed with AML and registered by the MIGICCL between 2019 and 2021.

**Gender [*n* (%)]**	***N* = 77**
Male	50 (64.9)
Female	27 (35.1)
**Age at diagnosis**	***N* = 77**
Range	0 m–17.9 years
Mean	9.4 years
**Age group [*n* (%)]**	***N* = 77**
Infants: <2 years	7 (9.1)
Children: 2 to <13 years	43 (55.8)
Adolescents: 13 to <18 years	27 (35.1)
**WBC count (×10^9^/L)**	***N* = 77**
Range	0.76–500
Mean	67.96
**FAB subtype [*n* (%)]**	***N* = 77**
M0	2 (2.6)
M1	6 (7.8)
M2	23 (29.9)
M3	14 (18.2)
M4	23 (29.9)
M5	2 (2.6)
M6	1 (1.2)
M7	6 (7.8)

WBC, white blood cells; FAB, French–American–British.

### Frequencies of fusion genes in child population with AML

The analyzed fusion genes showed a prevalence of 50.7% in our pediatric population (Table 2). *AML1-ETO* was the most frequent, with 22.1%, and was prevalent in the M2 subtype (39.2%) (Table 2). *PML-RARA* and *CBFB-MYH11* had the second and third frequency positions, with 20.8% and 5.2%, respectively (Table 2). *PML-RARA* showed the highest prevalence in M3 (85.7%) and *CBFB-MYH11* in M4 (8.7%) (Table 2). *BCR-ABL1^p210^* was detected with a frequency of 2.6% and was prevalent in M2 (4.3%) and M4 (4.3%), while *MLL-AF9* was not detected (Table 2). We did not detect the coexistence of fusion genes.

**Table 2:** Fusion gene frequencies and FAB subtypes of pediatric patients with AML.

FAB subtype^a^									
Fusion gene	M0	M1	M2	M3	M4	M5	M6	M7	Total^b^
*AML1-ETO*	–	3 (50%)	9 (39.2%)	–	4 (17.4%)	–	–	1 (16.7%)	17 (22.1%)
*PML-RARA*	–	–	1 (4.3%)	12 (85.7%)	3 (13.1%)	–	–	–	16 (20.8%)
*CBFB-MYH11*	–	–	1 (4.3%)	–	2 (8.7%)	1 (50%)	–	–	4 (5.2%)
*BCR-ABL1^p210^*	–	–	1 (4.3%)	–	1 (4.3%)	–	–	–	2 (2.6%)
*MLL-AF9*	–	–	–	–	–	–	–	–	–
*Not detected*	2 (100%)	3 (50%)	11 (47.9%)	2 (14.3%)	13 (56.5%)	1 (50%)	1 (100%)	5 (83.3%)	38 (49.3%)

^a^ Frequencies regarding FAB subtypes.

^b^ Frequencies regarding total samples.

### Fusion gene frequencies according to patients' age groups and FAB subtypes

The prevalence of fusion genes with respect to the three groups of age (see Methods) and FAB subtype was analyzed. As shown in Table 3, the children and adolescents were positive for four of the five fusion genes analyzed, while the infants did not show the presence of fusion genes. The children and adolescents had similar overall fusion gene frequencies of ∼55.7%; however, *AML1-ETO* showed a prevalence in children (27.9%), and *PML-RARA* was prevalent in adolescents (22.3%) (Table 3). The most common FAB subtypes in infants, children, and adolescents were M7, M2, and M4, respectively (Table 3). The FAB subtype M3 showed a similar distribution in children and adolescents, but it was not present in infants (Table 3).

**Table 3:** Fusion gene frequencies in pediatric patients with acute myeloid leukemia according to age group and FAB subtype.

		Fusion gene frequency			
Age group	Patients	Positives^a^	Type^a^	FAB^a^	Fusion gene frequency^b^
Infants	7/77 (9.1%)	0	NA	M1, M2, M6: 3 (42.9%)	Not detected
(<2 years)					
				M7: (57.1%)	Not detected
Children	43/77 (55.9%)	24 (55.8%)	*AML1-ETO*: 12 (27.9%)	M1: 3 (7%)	*AML1-ETO* (66.7%)
(2 to <13 years)			*PML-RARA*: 10 (23.3%)		
			*CBFB-MYH11*: 1 (2.3%)	M2: 15 (34.9%)	*AML1-ETO* (33.3%)
			*BCR-ABL1^p210^*: 1 (2.3%)		*PML-RARA* (6.7%)
					*BCR-ABL1^p210^* (6.7%)
				M3: 9 (20.9%)	*PML-RARA* (77.8%)
				M4: 12 (27.9%)	*AML1-ETO* (33.3%)
					*PML-RARA* (16.7%)
				M5: 1 (2.3%)	*CBFB-MYH11* (100%)
				M7: 1 (2.3%)	*AML1-ETO* (100%)
				M0: 2 (4.7%)	Not detected
Adolescents	27/77 (35%)	15 (55.6%)	*AML1-ETO*: 5 (18.5%)	M1: 2 (7.4%)	*AML1-ETO* (50%)
(13 to <18 years)			*PML-RARA*: 6 (22.3%)		
			*CBFB-MYH11*: 3 (11.1%)	M2: 7 (25.9%)	*AML1-ETO* (57.1%)
			*BCR-ABL1^p210^*: 1 (3.7%)		*CBFB-MYH11* (14.3%)
				M3: 5 (18.5%)	*PML-RARA* (100%)
				M4: 11 (40.7%)	*PML-RARA* (9.1%)
					*CBFB-MYH11* (18.2%)
					*BCR-ABL1^p210^* (9.1%)
				M5, M7: 2 (7.4%)	Not detected

NA, not applicable.

^a^ Frequencies regarding each age group.

^b^ Frequencies regarding FAB subtype.

Children constituted the largest group, with 55.9% of the patients and a fusion gene frequency of 55.8%, being positive for *AML1-ETO*, *PML-RARA*, *CBFB-MYH11*, and *BCR-ABL1^p210^* (Table 3). This group comprised patients with diverse subtypes, with M2 being the most prevalent (34.9%), followed by M4 (27.9%), and M3 (20.9%) (Table 3). *AML1-ETO* was the most prevalent in children, detected in M1 (66.7%), M2 (33.3%), M4 (33.3%), and M7 (Table 3). *PML-RARA* was the second most prevalent, which was detected in M2 (6.7%), M3 (77.8%), and M4 (16.7%) (Table 3). Finally, *CBFB-MYH11* and *BCR-ABL1^p210^* were the least frequent in children; *CBFB-MYH11* was solely detected in the sample of M5, while *BCR-ABL1^p210^* was detected in M2 with a frequency of 6.7% (Table 3). Adolescents constituted the second largest group, with 35% of the patients and a frequency of fusion genes of 55.6%, being positive for *AML1-ETO*, *PML-RARA*, *CBFB-MYH11*, and *BCR-ABL1^p210^* (Table 3). This group comprised subtypes M1–M5 and M7, where M4 was the most prevalent, with 40.7%, followed by M2 (25.9%) and M3 (18.5%) (Table 3). *PML-RARA* was the most prevalent in adolescents, which was detected in M3 (100%) and M4 (9.1%) (Table 3). *AML1-ETO* was the second most prevalent and was detected in M1 (50%) and M2 (57.1%) (Table 3). *CBFB-MYH11* was the third most prevalent in adolescents, and was present in M2 (14.3%) and M4 (18.2%) (Table 3). Finally, infants were the smallest group, with 9.1% of the patients, and they did not show the presence of fusion genes. This group comprised M1 (14.3%), M2 (14.3%), M6 (14.3%), and M7 (57.1%) sub-types (Table 3).

## Discussion

We report the prevalence of *AML1-ETO*, *PML-RARA*, *CBFB-MYH11*, *BCR-ABL1^p210^* and *MLL-AF9* in a Mexican pediatric population with newly diagnosed AML recruited between January 2019 and June 2021. This constitutes an effort of the MIGICCL to increase the knowledge and comprehension of the epidemiological behavior of these fusion genes within the childhood population of Mexico City, which has one of the highest incidence rates of acute leukemia in the world (3, 4).

Previous studies have reported a prevalence of the M2 (avg = 19.8%), M3 (avg = 21.4%), and M4 (avg = 16.6%) subtypes in certain Latin American populations, with a major susceptibility to M3 (4, 22). In agreement with these reports, we identified these three subtypes as prevalent in our population (M2 = 29.9%, M4 = 29.9%, and M3 = 18.2%); however, M3 was not the most common in this study.

The most frequent fusion genes detected were *AML1-ETO*, *PML-RARA*, and *CBFB-MYH11*, although *BCR-ABL1^p210^* was also detected at a lower frequency (Table 2). Altogether, these fusion genes account for a frequency of 50.7%, a percentage similar to that reported for pediatric patients around the world (6–9, 23). *AML1-ETO* was the most prevalent, which is consistent with previous reports that denote its ubiquity in patients with AML (23). However, our results showed a frequency that was ∼9% higher than that found in studies from the United States, Europe, Asia, and Australia, but it was 2.5% lower than that reported for the Japanese population (Supplementary Table S2). This fusion gene was prevalent in M2, but it was also detected in M1, M3, M4, and M7, in agreement with other studies, but there are no studies about their clinical impact (7–9, 24–27). *PML-RARA* had a prevalence that was ∼10% higher than that found in studies from the United Kingdom, Germany, Japan, and Austria, but not for Brazil, whose population has a 1.5% lower frequency (Supplementary Table S2). We found that this fusion gene was prevalent in M3, but it was also detected in M2 and M4 (Table 2), which has been reported as a rare event (28, 29). *CBFB-MYH11* showed a similar frequency to that found in studies from the United Kingdom, Brazil, and Japan (7), although studies from the United States, Germany, Austria, and Switzerland have reported frequencies as high as 11.3% (Supplementary Table S2). This fusion gene has been associated with the M4 subtype with abnormal eosinophils (M4Eo) (30), but it could also be detected in M4 without eosinophilic abnormalities, M2, and M5, as we observed in this study (Table 2). *BCR-ABL1* or the Philadelphia chromosome (Ph+) has been listed in the 2016 revised WHO classification of myeloid malignancies as a provisional entity (31). The incidence of Ph + in AML is approximately 0.5%–3%, however, an incidence of <1% was reported in studies with only purely *de novo* cases (12). Most of the patients with Ph+ express the *BCR-ABL1^p210^* transcript, while few cases express *BCR-ABL1^p190^* (12, 31). Although we analyzed these two transcript types, we only detected *BCR-ABL1^p210^*, which was four times more prevalent than reported for the population of the United Kingdom, but it was not detected in the Brazilian population (Supplementary Table S2). Due to the lack of studies with a pediatric population and the low prevalence of this fusion gene (12, 32), large-scale studies are needed to achieve better epidemiological/molecular characterization. Finally, although *MLL-AF9* is one of the most common types of *MLL* fusions in AML (11, 26) we did not detect it in our population (Table 2), probably due to the small number of infant samples analyzed. However, it has been reported that *MLL* can translocate with other genes, such as *AF10* and *ENL*, but we did not analyze it in this study. It is important to mention that, in a previous study on this same population, it was identified that children with acute leukemias treated in public hospitals are not genetically predominantly European, African, or Amerindian, but are a mixture of the three, so there are arguments that suggest that the population included in this study was a mixed-ethnicity population (33).

We performed a follow-up of patients to explore the relationship of fusion genes with mortality rates during the first year of treatment (Supplementary Figure S3). Several studies associate *AML1-ETO*, *PML-RARA*, and *CBFB-MYH11* with favorable prognosis and long survival; *BCR-ABL1^p210^* is associated with adverse prognosis, while *MLL-AF9* has been associated with intermediate to poor prognosis (23). Patients with *CBFB-MYH11* and *AML1-ETO* showed the highest rates of overall survival (Supplementary Figure S3), which is consistent with many previous reports indicating long survival and low mortality rates for these patients (23). In this study, patients with *CBFB-MYH11* did not die, and only one of the *AML1-ETO* patients died (Supplementary Table S1). One of the two patients with *BCR-ABL1^p210^* died, which was an expected outcome due to the association of these fusion genes with adverse prognosis (34). Although this is most likely not because of *BCR-ABL1* itself but rather due to other high-risk cytogenetic/molecular features that are present in the majority of cases (35), it is important to note that we only had two positive patients, which makes it difficult to draw conclusions about the impact of this fusion gene in our population (Table 2). It has been reported that 70%–80% of patients with newly diagnosed M3 carrying *PML-RARA* achieve long-term remission. However, mortality in the first 30 days following therapy remains a major contribution to treatment failures, which is mainly attributable to thrombotic or hemorrhagic complications, reporting early mortality rates of 3%–20% (36–38). In this study, patients with *PML-RARA* had an early mortality rate of 18.75% due to intracranial hemorrhages (3/16 patients), showed that all of the deceased patients died within the first months after diagnosis (Supplementary Table S1). This early mortality rate shown in our population could be an overestimate due to the number of patients analyzed in comparison with other studies, or an indication of aspects related to local protocols that need improvement (36–39). Further studies are needed to establish an association.

A limitation of this study was the use of conventional PCR to detect *AML1-ETO*, *PML-RARA*, *CBFB-MYH11*, *BCR-ABL1^p210^* and *MLL-AF9*. However, we are performing a prospective study using Real-Time Quantitative PCR to increase the power detection of these fusion genes, as well as the incorporation of primers and probes to detect the main variants of *CBFB-MYH11*, the *BCR-ABL^p190^* and *MLL-ENL* fusion gen.

The use of molecular diagnostics to detect fusion genes with an impact on therapy has contributed to improved survival rates for pediatric patients with AML in developed countries (2). However, fusion gene screening by molecular approaches is not a routine activity in most public hospitals in Mexico City; nevertheless, it may be available through research projects such as this study. Besides, it is very important that fusion gene screening can be implemented with standard criteria in all public hospitals where children with AML are cared for. In this way, it may be possible to improve the survival rates in our pediatric population. Finally, we believe that long-term follow-up studies are needed to support solid conclusions about the impact of fusion genes on the survival of Mexican pediatric patients with AML.

## Conclusions

The pediatric population of Mexico City with *de novo* AML had frequencies of *AML1-ETO*, *PML-RARA*, *CBFB-MYH11*, and *BCR-ABL1^p210^* similar to those of other populations around the world. The patients with *BCR-ABL1^p210^* and *CBFB-MYH11* were few or did not die, while those with *MLL-AF9* was not detected in this study. Although patients with *PML-RARA* had a low survival and a high early mortality rate, further studies are needed to determine the long-term impacts of these fusion genes on this Latino population.

## Data Availability

The original contributions presented in the study are included in the article/**Supplementary Material**, further inquiries can be directed to the corresponding author/s.
