# Supplementary material for: Analytical study of RUNX1-RUNXT1, PML-RARA, CBFB-MYH11, BCR-ABL1p210, and KMT2-MLLT3 in Mexican children with acute myeloid leukemia: A multicenter study of the Mexican interinstitutional group for the identification of the causes of childhood leukemia (MIGICCL)
Source: Front Pediatr. 2022 Nov 14;10:946690. doi: 10.3389/fped.2022.946690 (PMC9702800; doi:10.3389/fped.2022.946690)
Supplement: Supplementary file 1 [file Datasheet1.docx]

**Supplementary Material**

**Supplementary Figure 1.** Electrophoresis of PCR products of some representative patients detected as positive for fusion genes. M: Molecular marker Gene Ruler 100 bp DNA Ladder.

**
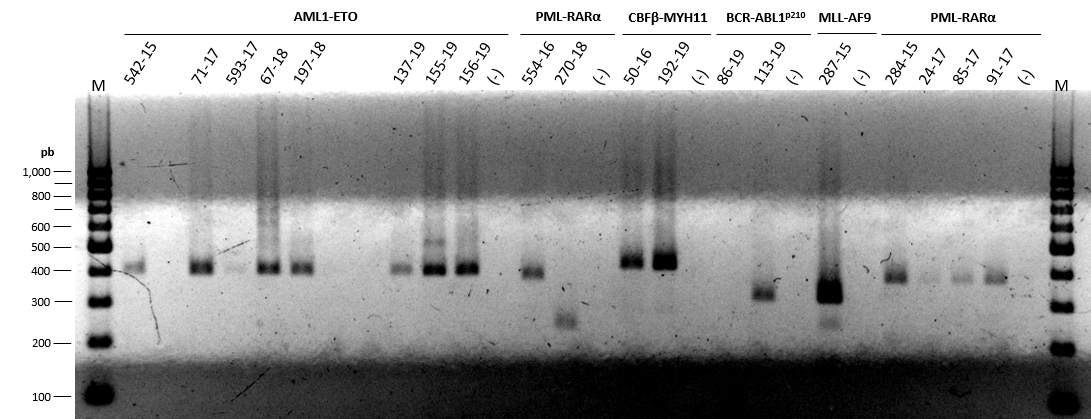
**

**Supplementary Figure 2.** DNA sequences of PCR products from representative positive samples for fusion genes**.** Sequence segments for each gene are marked with color red and green. Primers sequences are indicated with underlined text. *bcr^1,2,3^*: break point region 1, 2, or 3.

**Supplementary Figure 3.** Kaplan-Meier analysis of overall survival in pediatric patients with AML according to the presence of fusion genes.


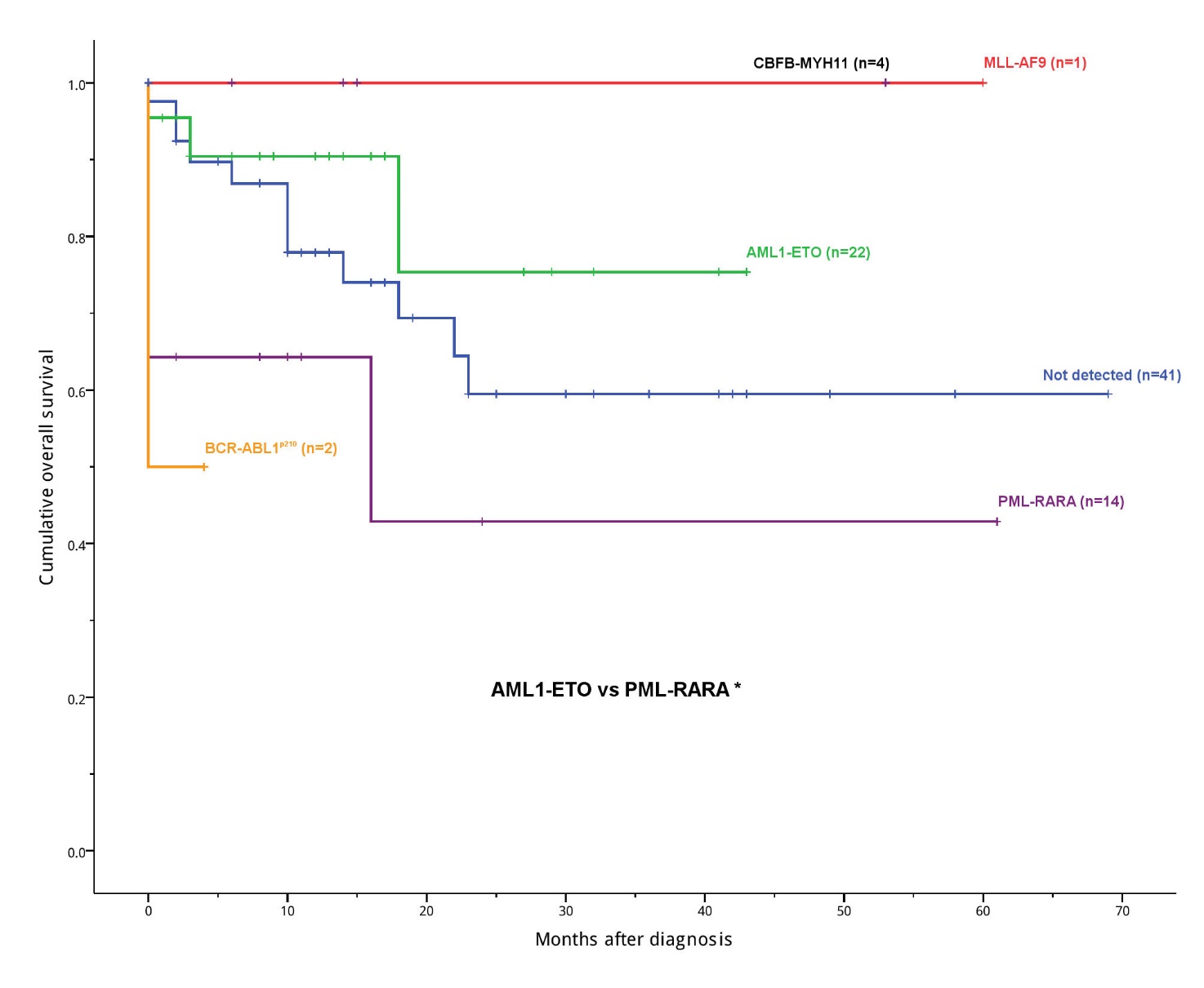


*: Significant statistical differences (*p=0.035*).

**Supplementary Table 1.** Description of deceased patients according to fusion genes and *PML-RARA* alive patients.

|  | **Deceased patients** | | |
| --- | --- | --- | --- |
| **Fusion gene and breakpoint cluster region (*bcr*)** | Cause and time | FAB subtype | Treatment |
| *AML1-ETO* |  |  |  |
| *-NA* | 1. Pneumonia; 3 months | M2 | NOPHO-AML93 |
| *PML-RARα* |  |  |  |
| *-bcr^1-2^* | 1. Intracranial hemorrhage; < 1 months | M3 | PETHEMA-APL05 |
| *-bcr^1-2^* | 1. Intracranial hemorrhage; < 1 months | M3 | PETHEMA-APL05 |
| *-bcr^3^* | 1. Intracranial hemorrhage; < 1 months | M4 | BFM-2001 |
| *-bcr^1-2^* | *Alive* | M3 | PETHEMA-APL05 |
| *-bcr^1-2^* | *Alive* | M4 | BFM-2001 |
| *-bcr^1-2^* | *Alive* | M4 | BFM-2001 |
| *-bcr^1-2^* | *Alive* | M3 | PETHEMA-APL05 |
| *-bcr^1-2^* | *Alive* | M3 | PETHEMA-APL05 |
| *-bcr^1-2^* | *Alive* | M3 | PETHEMA-APL05 |
| *-bcr^1-2^* | *Alive* | M3 | BFM-1998 |
| *-bcr^1-2^* | *Alive* | M3 | PETHEMA-APL05 |
| *-bcr^1-2^* | *Alive* | M3 | PETHEMA-APL05 |
| *-bcr^1-2^* | *Alive* | M3 | BFM-1998 |
| *-bcr^3^* | *Alive* | M3 | PETHEMA-APL05 |
| *-bcr^3^* | *Alive* | M3 | PETHEMA-APL05 |
| *-bcr^3^* | *Alive* | M3 | PETHEMA-APL05 |
| *BCR-ABL1^p210^* |  |  |  |
| *-NA* | 1. Hemorrhage; < 1 months | M4 | NOPHO-AML93 |
| *Not detected* |  |  |  |
| *-NA* | 1. Septic shock; < 1 month | M7 | BFM-1998 |
| *-NA* | 1. Disseminated intravascular coagulation; 10 months | M4 | BFM-1998 |
| *-NA* | 1. Other; 10 months | M4 | NOPHO-AML93 |
| *-NA* | 1. Septic shock; 6 months | M6 | PETHEMA-APL05 |
| *-NA* | 1. Intracranial hemorrhage; 10 months | M4 | BFM-2001 |
| *-NA* | 1. Septic shock; 1 month | M4 | BFM-1998 |
| *-NA* | 1. Septic shock; < 1 month | M2 | BFM-1998 |
| *-NA* | 1. Other; 2 months | M2 | BFM-1998 |
| *-NA* | 1. Septic shock; < 1 month | M0 | BFM-1998 |

NA: not applicable.

**Supplementary Table 2.** Fusion gene frequencies in previous studies.

|  | **Reference/Age (years)/Country ^a^** | | | | | | | |
| --- | --- | --- | --- | --- | --- | --- | --- | --- |
|  | (7) | (8) | (9) | (23) | (24) | (25) | (26) | This study |
|  | <14 | <16 | <18 | <18 | <11 | <18 | <22 | <18 |
| **Fusion gene** | GB | GB | DE, AT, CH, CZ | DE, AT, CH | BR | JP | US | MX |
| *AML1-ETO* | 12% | 13.6% | 12.6% | 12-14% | 10.4% | 25.5% | 13.5% | 22.1% |
| *PML-RARα* | 9.1% | † | 5.9% | 6-10% | 18.5% | 8% | † | 20.8% |
| *CBFβ-MYH11* | 4.7% | 6.8% | 9.2% | 8% | 5.1% | 6.2% | 11.3% | 5.2% |
| *BCR-ABL1^p210^* | 0.6% | ‡ | ‡ | ‡ | 0 | ‡ | ‡ | 2.6% |
| *MLL-AF9* | 2% | 6.3% | 9.9% | 7% | 2.8% | 7.4% | 6.5% | 0 |

†: Patients with diagnosis of acute promyelocytic leukemia was excluded.

‡: Not analyzed.

a: ISO Country Codes, GB (United Kingdom), US (United State), DE (Germany), AT (Austria), CH (Switzerland), CZ (Czech Republic), BR (Brazil), JP (Japan), MX (Mexico).

7. (Grimwade, D., 2001): 340 children entered into the MRC AML10 trial for children over 8-years period (1988-1995).

8. (Harrison, C.J., et al. 2010): 729 children entered onto the United Kingdom Medical Research Council AML trials over 15-years period (1988-2002).

9. (von Neuhoff, C., et al. 2010): 454 children enrolled in the AML-BFM 98 trial over 7-years period (1998-2004).

23. (Creutzig, U., et al. 2012): Not specified the number of children with AML

24. (Andrade, F.G., et al. 2016): 703 children enrolled over 16-years period (2000-2015).

25. (Iijima-Yamashita, Y., et al. 2018): 448 children enrolled in the JPLSG AML-05 study over 5-years period (2006-2010).

26. (Rubnitz, J.E., et al. 2010): 206 children enrolled over 7-years period (2002-2008).

(This study): 77 children enrolled over 3-years period (2019-2021).
